# Severe Lower Limb and Abdominal Edema Associated with Subcutaneous Apomorphine Infusion

**DOI:** 10.1002/mdc3.70681

**Published:** 2026-05-13

**Authors:** Guillaume Costentin, David Maltête

**Affiliations:** ^1^ Department of Neurology, Rouen University Hospital University of Rouen Rouen France; ^2^ Inserm U1239, Laboratory of Neuronal and Neuroendocrine Differentiation and Communication Mont‐Saint‐Aignan France

**Keywords:** apomorphine, edema, Parkinson's disease

Subcutaneous apomorphine infusion represents a well‐validated therapeutic option for Parkinson's disease patients experiencing motor fluctuations refractory to optimized oral pharmacotherapy. Its efficacy and tolerability have been observed in numerous studies.^1^ Well‐known adverse effects include nausea, orthostatic hypotension, and cutaneous reactions—particularly injection‐site nodules, which affect up to 70% of patients and may cause itching, pain, burning, or even ulceration. We report a very rare case of a patient who developed severe edema following apomorphine initiation, with complete resolution upon discontinuation.

A 60‐year‐old woman with a history of non‐insulin‐dependent diabetes, sleeve gastrectomy, and surgically treated breast cancer was diagnosed with Parkinson's disease in 2019, presenting with asymmetric akinesia‐rigidity and right‐sided tremor. Due to motor fluctuations, morning dystonia, and mild dyskinesia, daytime subcutaneous apomorphine infusion was initiated during hospitalization. The dose was gradually titrated to 4 mg/h, with good initial tolerance. However, 12 days after initiation, she presented to the emergency department with progressive, pitting edema of the lower limbs extending to the abdomen (Fig. 1). There were no signs of heart failure, dyspnea, or abnormal blood pressure, and no clinical signs of inflammation that would have suggested cellulitis due to local diffusion of apomorphine. Laboratory tests revealed no electrolyte imbalance, inflammation, eosinophilia, or hepatic dysfunction. Apomorphine was discontinued, leading to rapid improvement and complete resolution of edema within 3 days.

**Figure 1:**
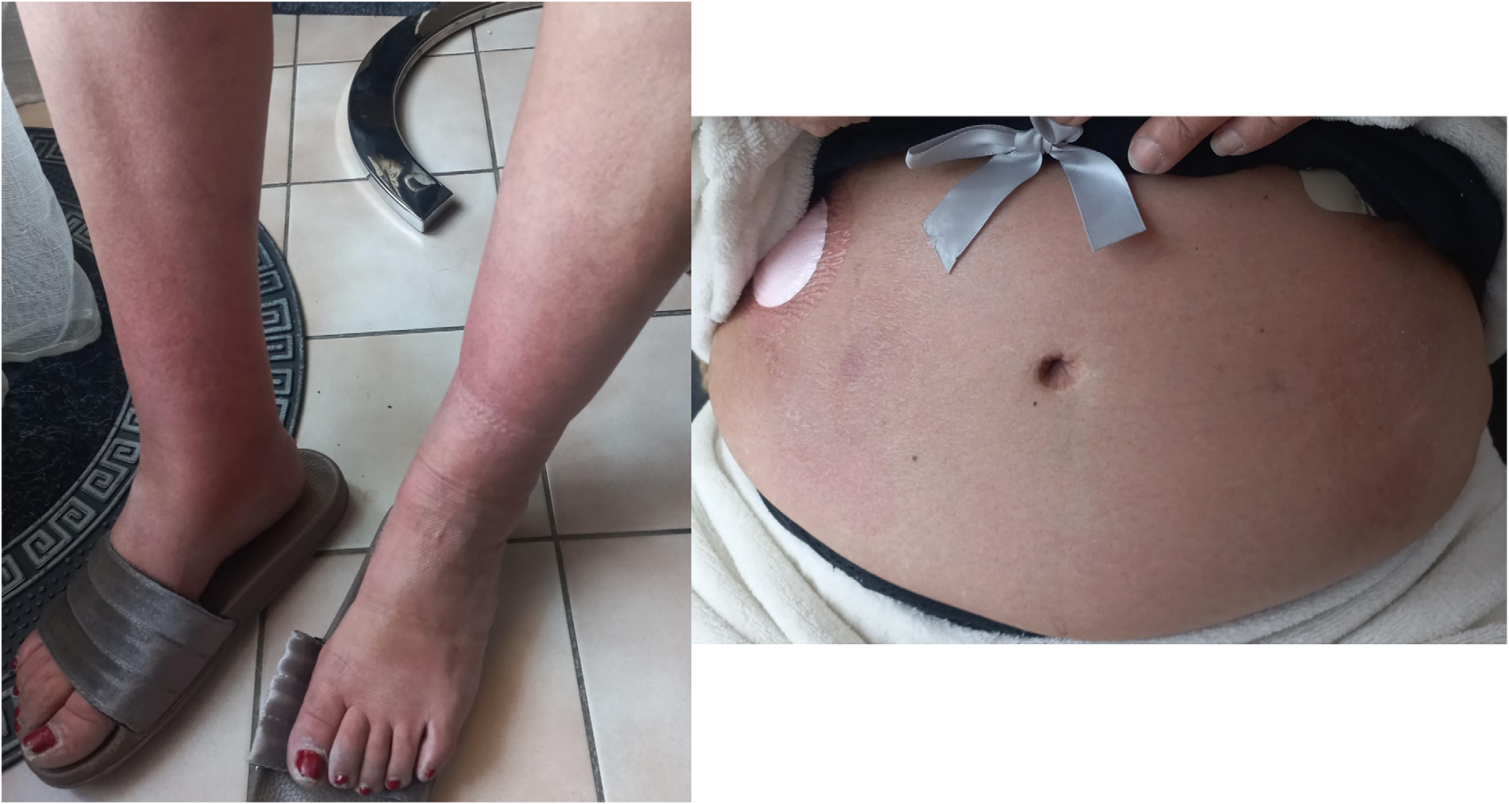
Clinical photographs demonstrating severe edema of the lower limbs and abdomen.

Dopamine agonists, including apomorphine, are known to cause peripheral edema, with reported frequencies ranging from 5% to 22%. Risk factors include coronary artery disease, diabete and female sex,^2^ both of which were present in our patient. Dopamine agonist induced edema is typically localized to the lower extremities, is dose‐dependent and diuretic‐resistant, and recurs upon reintroduction.

To date, only one case report^3^ describes severe apomorphine‐induced edema: a 55‐year‐old man developed progressive lower limb edema and a 9‐kg weight gain 10 days after apomorphine initiation (5 mg/h). There was no evidence of cardiac etiology, and edema resolved within 2 days of discontinuation. Rechallenge at a lower dose (3 mg/h) led to recurrence.

The underlying mechanisms remain poorly understood. Both ergoline and non‐ergoline agonists may cause peripheral edema through complex peripheral nervous system interactions. Stimulation of D1 receptors induces arteriolar vasodilation, increasing capillary hydrostatic pressure.^4^ D2‐like receptor activation indirectly causes vasodilation by inhibiting sympathetic vasoconstrictor activity. Central D2 receptor stimulation reduces peripheral norepinephrine, further decreasing arteriolar vasoconstriction and venous tone.^5^

Renal dysregulation also plays a role. Renal dopamine, synthesized in proximal tubules from L‐dihydroxyphenylalanine, inhibits the Na+/K+ pump, promoting natriuresis. Agonists, which are renally excreted, may compete with dopamine for cationic transporters, reducing sodium excretion and promoting edema.^6^

The rarity of reported apomorphine‐induced edema cases may reflect observation bias, as pramipexole is more widely prescribed.^7^ Pharmacokinetic differences may also contribute: pramipexole is excreted unchanged in urine, directly impacting natriuresis, whereas apomorphine is rapidly metabolized to inactive compounds, reducing renal exposure and tissue receptor stimulation.

This case highlights the potential for severe, generalized edema with subcutaneous apomorphine infusion, a rarely reported but clinically significant adverse effect. Clinicians should be aware of this possibility, especially in patients with risk factors, and consider prompt discontinuation if edema develops.

## Author Roles

(1) Research project: A. Conception, B. Organization, C. Execution; (2) Manuscript Preparation: A. Writing of the first draft, B. Review and Critique.

G.C.: 1A, 1B, 1C, 2A, 2B

D.M.: 1A, 1BC, 2B

## Disclosures

**Ethical Compliance Statement:** According to institutional policies, ethical approval from an Institutional Review Board was not required for this single case report. Written informed consent was obtained from the patient for publication of this case report and any accompanying images. The consent form is held by the authors and is available for review by the Editor if required. We confirm that we have read the Journal's position on issues involved in ethical publication and affirm that this work is consistent with those guidelines.

## Financial Disclosures and Conflicts of Interest

Author disclosures are available in the Supporting Information.

## Supporting information


**Data S1.** ICMJE both authors.

## Data Availability

The data that support the findings of this study are available on request from the corresponding author. The data are not publicly available due to privacy or ethical restrictions.
